# Influence of the Hubbard *U* Parameter on the Structural, Electronic, Magnetic, and Transport Properties of Cr/Fe/Zr-Based MBenes

**DOI:** 10.1021/acsomega.3c06539

**Published:** 2023-11-16

**Authors:** Isabel M. Arias-Camacho

**Affiliations:** Faculty of Physics, University of Warsaw, Pasteura 5, Warsaw PL-02-093, Poland

## Abstract

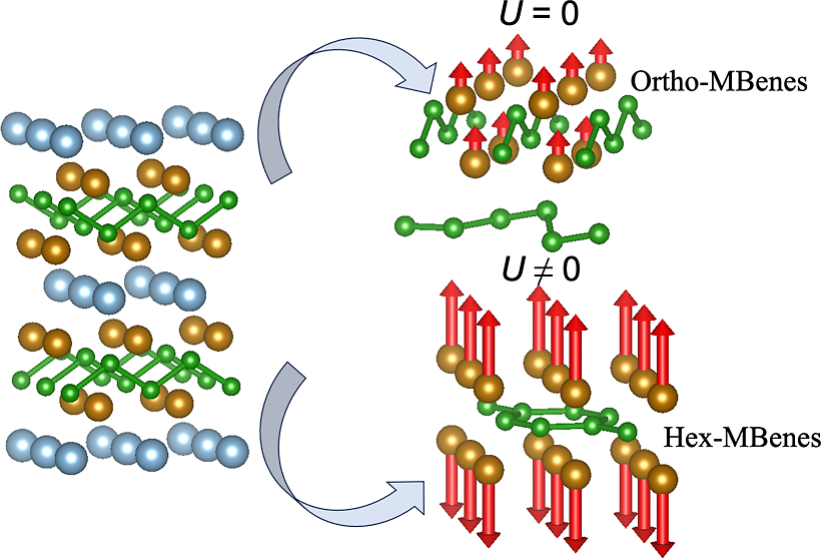

Although relatively new, MBenes are gaining prominence due to their outstanding mechanical, electronic, magnetic, and chemical properties, and they are predicted to be good electrodes for catalytic processes as well as robust 2D magnets with high critical temperatures, to mention some of their intriguing attributes. From all their multiple stoichiometries, a theoretical study of their orthorhombic and hexagonal phases in the framework of density-functional theory is performed in this work. The results suggest that their properties are strongly dependent on the initial conditions considered in the theoretical approach and must be treated with caution. However, and independently of these factors, all of them are demonstrated to be energetically stable, show a metallic behavior, and exhibit, in specific cases, large magnetic moments per unit cell, exceeding 6.5 μ_B_ in the case of the orthorhombic-type Cr_2_B_2_, making them suitable as robust 2D magnets with room critical temperature. These findings represent an important step toward a better understanding of MBenes, opening several windows to future research in energy conversion and storage, sensing, catalysis, biotechnology, or spintronics.

## Introduction

The purpose of this work is to understand, with a computational approach, the origin of the structural, electronic, magnetic, and transport properties of selected transition metal (TM) monoborides, also known as MBenes (M = Cr, Fe, and Zr), as well as to identify features that may affect the transport properties of these compounds. All these MBenes, some of them already proven to be stable by previous study,^1^ present good structural stability and some of them exhibit robust magnetism, as well. 2D MBenes are present in a variety of stoichiometries (M_2_B_2_, M_2_B_3_, and M_3_B_4_). The chosen structures for the MBenes described here possess M_2_B_2_ stoichiometry, which can be either orthorhombic (in the following, ortho-MBenes) with *Pmma* (no. 51) space group symmetry or hexagonal (in the following, hex-MBenes) with *P*6/*mmm* (no. 191) space group symmetry. In the *Pmma* structures, each atom is surrounded by six neighbors, and the buckled boron bilayers are sandwiched between the TM layers. On the other hand, in the *P*6/*mmm* structures, the honeycomb, graphene-like, boron layer is sandwiched between two TM layers on both sides, with every TM atom located above or below the centroid of the honeycomb structure. Their bulk counterparts, all widely studied both experimentally and theoretically,^2–4^ are the ferromagnetic (FM) α and β modifications of FeB and the nonmagnetic (NM) CrB and ZrB compounds. The structure of α-FeB is debatable,^4^ whereas β-FeB and CrB are orthorhombic crystals with *Pnma* (no. 62) and *Cmcm* (no. 63) space group symmetries, respectively. The ZrB solid is rock-salt structured and crystallizes in cubic *Fm*3̅*m* (no. 225) space group symmetry. The β-FeB and CrB solids exhibit very interesting structures since both enclose boron double-chain stripes which are very common motives of all-boron nanostructures.^5,6^ Parallel to MAX phases in MXenes, MBenes can be obtained by removing the element A by chemical etching from their parental MAB phases. Ade and Hillebrecht^7^ were the first to identify MBenes as derivatives of MXenes in 2015. Since then, a lot of efforts have been done in the search of boron-based 2D materials with excellent charge carrier mobility, versatile chemical activity, magnificent specific surface area or good mechanical strength, well-desired characteristics in a low-dimensional material. Although relatively young and in process of being explored,^8^ synthesized,^9^ and understood, the TM monoborides present a high potential in diverse applications like energy conversion and storage,^10^ catalysis,^11,12^ NO electroreduction,^13^ adsorption and activation of CO_2_,^14^ biotechnology,^15^ magnetic refrigeration,^16^ information storage devices,^17^ or spintronics,^18–21^ among others. It was thought for several years that the long-range magnetic order was not possible in 2D materials due to thermal fluctuations.^22^ However, contradicting the predictions of the Mermin–Wagner theorem, the magnetic anisotropy energy originating on the spin orbit coupling can shield this effect, confirming consequently the existence of 2D magnets.^23^ Nevertheless, most of them, like CrI_3_^24^ or Fe_3_GeTe_2_,^25^ show low critical temperatures (≈45 and 130 K, respectively), reason why the achievement of materials with higher critical temperatures remains a challenge for their use in magnetic storage devices and spintronics. In the recent years, several research studies involving MBenes point in this direction.^18,20^ Another issue to deal with is the consideration (or not) of the correction introduced by the Hubbard parameter, *U*, which accounts appropriately for the strongly correlated electrons in the d orbitals of the TMs. Both approaches are found in the literature, almost equally, when diverse properties of the 2D TM monoborides are studied. However, the differences in the results of both methodologies can lead to completely different conclusions, for example, in the final magnetic ground state (MGS) or the most stable geometry, which in specific cases become the opposite the one to the other. Then, a correct treatment of the initial premises seems not obvious.^20^ Following these premises, this paper intends to elucidate how the different mechanisms influence in the determination of the physical and chemical properties, crucial in the search of further technological applications. This work is structured in the following parts. In the first section, the structures of the selected MBenes are computed to determine the Hubbard parameters for each of them based on their optimized geometries and MGSs. Once obtained, the stability, conductivity, and electronic and magnetic properties will be compared and discussed in both contexts: with and without the correction, to end with the main conclusions and a view into their future applications.

## Computational Approach

In the framework of the density-functional theory (DFT), first-principles spin-polarized calculations have been performed, within the generalized gradient-corrected approximation of Perdew–Burke–Ernzerhof (PBE)^26^ for the exchange–correlation functional, using projector plane-wave pseudopotentials^27^ implemented in the Quantum ESPRESSO (QE) suite of codes.^28^ Every unit cell contains two atoms of the TM and two atoms of boron, with an empty space of thickness of 15 Å along the normal direction to avoid interactions between adjacent MBenes. The optimization of geometries has been done, allowing the unit cell shape, volume, and the ions to relax until the residual forces on the atoms have been less than 0.3 meV/Å and the total energy convergence has been set to 10^–5^ Ry. The electronic wave functions and the charge density have been expanded in plane-wave basis sets with energy cutoffs of 70 and 700 Ry, respectively, while the Γ-centered *k*-point grid in the Brillouin zone, in the Monkhorst–Pack scheme, has been set to 12 × 12 × 1 for the geometry optimizations and 24 × 24 × 1 for the density of state (DOS) calculations, with a Gaussian smearing of 0.02 Ry; the accuracy of the total energy is ensured by these values. To determine the most energetically favorable MGS of each structure, two collinear calculations, one FM and one antiferromagnetic (AFM), have been used. The geometry relaxations of the 2D structures have been performed with PBE, and the magnetic and electronic properties have been calculated using both PBE and PBE+*U*. An important descriptor used for the structural characterization is the cohesive energy per atom (*E*_coh_), that is, the difference in energy between the total energy of the compound and the sum of the total energies of the isolated atoms
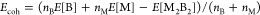
1which is the released energy when a compound dissociates into isolated free atoms, where M represents the TM atom, *E*[M_2_B_2_] is the total energy of the MBene, *E*[B] and *E*[M] are the total energies of the isolated atoms (B and TM atoms), and *n*_B_ and *n*_M_ are the numbers of boron and TM atoms per unit cell, respectively, directly obtained from the spin-polarized calculations. The transport integrals have been computed using the Boltzmann transport theory^29^ and a constant scattering rate model (the inverse of relaxation time was taken to be 0.1 eV), and a Bader analysis has been used to obtain the charge transfer. The visualizations have been performed using the Visualization for Electronic and STructural Analysis (VESTA) software.^30^

## Results and Discussion

### Structure

**Figure 1:**
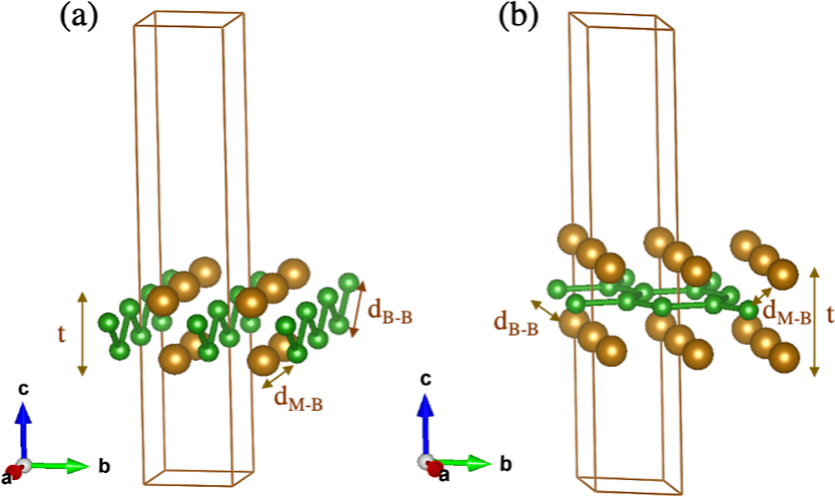
(a) Ortho-MBene and (b) hex-MBene structures of M_2_B_2_ corresponding to *Pmma* and *P*6/*mmm* symmetries, respectively. The unit cells used in the calculations are shown in brown.

It is expected that a mixture between boron and TM atoms will stabilize the structures since boron is electron deficient. Among all the possible stoichiometries of MBenes, we have focused on the orthorhombic (ortho-) and hexagonal (hex-) M_2_B_2_ structures, as shown in Figure 1.

In our previous work,^31^ we have performed a full structural optimization to determine the structural parameters of our unit cells, finding that the unit cells of ortho-MBenes become almost rectangular with *a* > *b* when the TM is Fe or Cr (*a*/*b* is 1.005 and 1.013 for chromium and iron, respectively), whereas *a* < *b* for ortho-Zr_2_B_2_ (*a*/*b* = 0.94). The lattice parameters, *a* = 2.885 and *b* = 2.870 Å for ortho-Cr_2_B_2_, *a* = 2.823 and *b* = 2.787 Å for ortho-Fe_2_B_2_, and *a* = 3.084 and *b* = 3.281 Å for ortho-Zr_2_B_2_; *a* = *b* = 2.919 Å for hex-Cr_2_B_2_, *a* = *b* = 2.913 Å for hex-Fe_2_B_2_, and *a* = *b* = 3.159 Å for hex-Zr_2_B_2_, have been compared to and are in good agreement with the values found in the literatures.^11,14,17,19,32–34^ The distances between the TMs and the boron atoms are always larger for the hexagonal systems (ranging between 2.15 and 2.49 Å) than those for the orthorhombic systems (between 2.04 and 2.46 Å), with the bigger distances corresponding to Zr_2_B_2_ MBenes.

### Determination of the *U* Parameter

The Hubbard *U* parameter is introduced in highly correlated systems due to the fact that LDA or GGA inadequately treats the self-interaction of the partially occupied Kohn–Sham (KS) orbital. In solids, it is even more complicated because the hybridization of localized orbitals can produce fractional occupations, causing the total energy to contain such effects from hybridization. In this work, we determine the Hubbard parameters for the TM d orbitals using the linear response approach^35^ based on the density-functional perturbation theory (DFPT).^36,37^ Within this framework, the Hubbard parameters can be computed from the second-order derivative of the energy. The total energy as a function of the localized orbital occupation *q*_*I*_ of Hubbard site *I* is given by
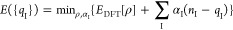
2where ρ is the charge density and α_I_ is the Lagrange multiplier (that acts as a perturbation potential) employed to constrain the site occupation *n*_I_ which is the occupation of the localized states in the d orbital of site I. It is more convenient to work with the Legendre transform of eq 2, which leads to a modified energy functional that depends on {α_I_}
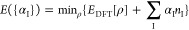
3

Then, the total energy as a function of on-site occupations *n*_I_ is given via a Legendre transform
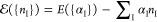
from which the second derivative can be evaluated with
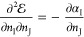
4

Similarly, the second derivative of the total energy for the noninteracting system evaluated through KS equations can be obtained from
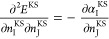
5

The effective interaction parameter *U* of site *I* can be calculated as a difference of the above-defined second derivatives of the energy of the interacting and noninteracting systems with respect to electronic occupation
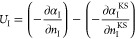
6

This approach to compute the Hubbard *U* parameters based on DFPT is implemented in QE in the HP code.^38,39^

Table 1 describes the values for Cr_2_B_2_ and Fe_2_B_2_ that are higher than those for Zr_2_B_2_. Such values of *U* are found to be similar to other works,^20,21^ so the consistency of these results is assumed. However, in order to perform a suitable comparison using the same parameter for both MGSs, the values of the second column (FM) were chosen as a criterion.

**Table 1:** Hubbard Parameters Calculated Using DFPT^a^

	FM		AFM	
MBene	*U*_TM1_	*U*_TM2_	*U*_TM1_	*U*_TM2_
ortho-Cr_2_B_2_	4.297	4.297	6.682	6.806
hex-Cr_2_B_2_	5.000	5.000	4.995	4.995
ortho-Fe_2_B_2_	4.346	4.279	4.651	4.556
hex-Fe_2_B_2_	4.232	4.231	4.009	4.010
ortho-Zr_2_B_2_	1.792	1.792	1.792	1.792
hex-Zr_2_B_2_	1.802	1.802	1.802	1.802

a FM and AFM indicate the ferro- and antiferromagnetic ground states, respectively, and TM1 and TM2 correspond to each transition metal in the unit cell.

### Ground-State Energetics

A high cohesive energy, *E*_coh_, indicates a high bond strength and hence a good thermodynamic stability. The cohesive energies of all the systems involved here have been calculated using eq 1. The corresponding results are shown in the second column of Table 2.

**Table 2:** Cohesive Energies (*E*_coh_) for the Orthorhombic and Hexagonal Structures of Cr_2_B_2_, Fe_2_B_2_, and Zr_2_B_2_

	*E*_coh_	
MBene	*U* = 0	*U* ≠ 0
ortho-Cr_2_B_2_	**6.222**	4.539
hex-Cr_2_B_2_	6.201	**4.558**
ortho-Fe_2_B_2_	**6.901**	6.235
hex-Fe_2_B_2_	6.830	**6.264**
ortho-Zr_2_B_2_	8.050	7.579
hex-Zr_2_B_2_	**8.087**	**7.615**

All our MBenes exhibit large cohesive energies ranging from 6.222 to 8.087 eV if *U* = 0 and from 4.466 to 7.178 eV after using the corresponding value of *U*, and they are similar to those found in the literature.^40^ All of them present strong internal binding and good stability, although, in general, the cohesive energies for *U* = 0 are always higher. To check the consistency of our values, we have computed the carbon diamond structure with the same parameters used in the optimization, resulting an *E*_coh_ of 7.757 eV, comparable to other experimental and theoretical work.^41^ Moreover, Zhang et al.^42^ reported a cohesive energy of 6.30 eV for ortho-Cr_2_B_2_, which is close to our 6.22 eV when *U* = 0.

The dependence of the structure stability with the atomic mass of the TM observed earlier^33^ is also present in our results, being hex-Zr_2_B_2_ the MBene with the highest *E*_coh_ (8.087 eV). Moreover, in a previous work,^31^ we have calculated the phonon dispersion of all the orthorhombic and hexagonal stoichiometries which have resulted dynamically stable with frequencies over 740 cm^–1^, in agreement to other studies.^13,21,34^

Interestingly, a remarkable difference between the two approaches employed is observed between the most energetically favorable geometries (which have been indicated in bold for more clarity): under the approximation with *U* = 0, Cr_2_B_2_ and Fe_2_B_2_ prefer orthorhombic configurations, as other works also predict,^1,32,43^ whereas Zr_2_B_2_ suits better the hexagonal structure from the point of view of the cohesive energy. The picture changes when *U* ≠ 0, for which all the structures become preferably hexagonal, indicating that the introduction of the correction has a strong influence on the stability. The existing attempts to obtain MBenes from ternary MAB phases have used ortho-MAB phases as precursor to synthesize MoB and CrB ortho-MBenes, with the result of poor quality, due to the complete dissolution of the parent phases or the partial etching of Al.^7,44–47^ On the other side, new attempts are focused on the fabrication of hex-MAB phases as a promising phase toward hex-MBenes.^48^ In this sense, up to date, there are scarce experimental results that can firmly confirm the adoption of the one or the other structure, and big efforts into this direction are still ongoing.^49,50^

It should be noted that both orthorhombic and hexagonal structures are very close in energy (some tens of eV), and investigations based on the nudged elastic band method have determined that the small energy barrier, between 0.2 and 0.4 eV per atom, could lead to the transformation of the ortho-MBenes into hex-MBenes at high temperatures.^1,10^

### Electronic and Transport Properties

**Figure 2:**
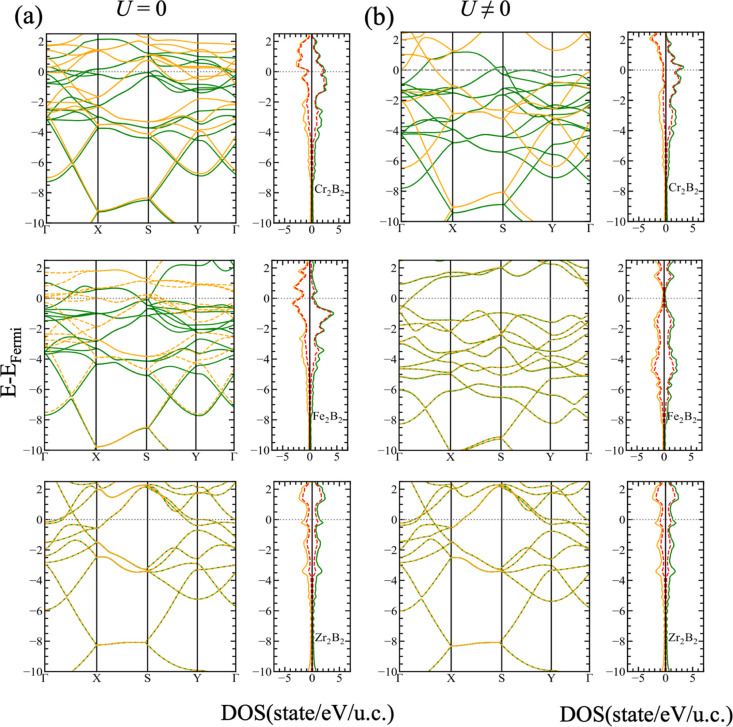
Comparison between the electronic band structure and DOS for the majority (green) and minority (orange) spins of the *Pmma* structures of Cr_2_B_2_, Fe_2_B_2_, and Zr_2_B_2_ using (a) DFT and (b) DFT+*U*. The dotted red line indicates the contribution of the TM-d orbitals.

The calculated spin-polarized band structures, DOSs, and projected densities of states (PDOSs) including *U* = 0 and *U* ≠ 0 are shown in Figures 2 and 4 for both orthorhombic and hexagonal structures, respectively. As expected,^9,11,21,40^ the behavior of these MBenes is always metallic, with no band gaps between the valence band and the conduction band, but with partially occupied bands crossing the Fermi level for the majority and minority spin channels. For clarity, the d states of the TMs have been plotted together with the total DOS, highlighting their major contribution at the Fermi level. The effect of introducing *U* becomes clearly visible after a comparison of the respective spin-polarized band structures, where the Coulombian repulsion expected by the introduction of the term increases the separation of the bands in the surroundings of the Fermi level.

**Figure 3:**
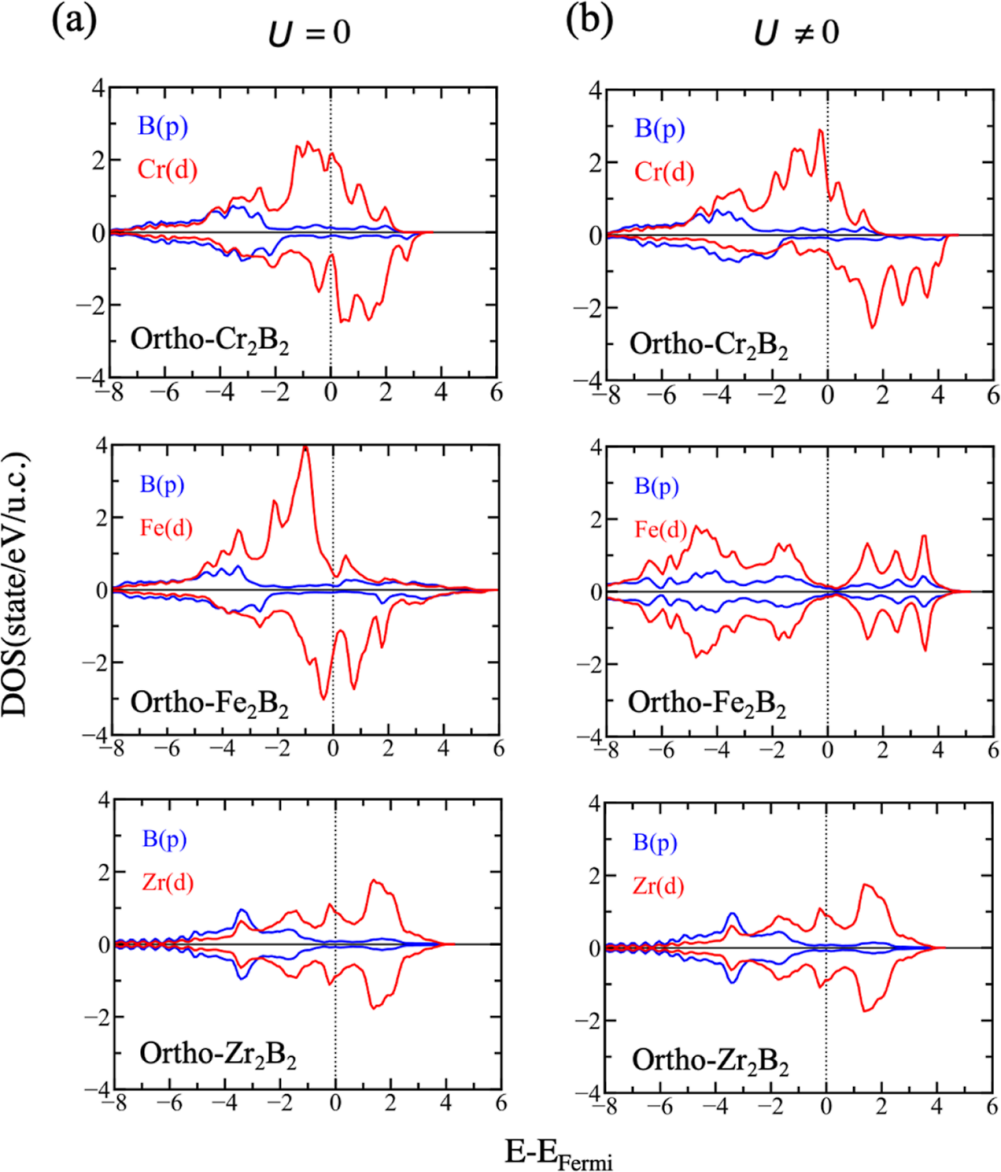
PDOS corresponding to the d states of the TM (red) and the p states of boron (blue) of the *Pmma* structures of Cr_2_B_2_, Fe_2_B_2_, and Zr_2_B_2_ using (a) DFT and (b) DFT+*U*.

**Figure 4:**
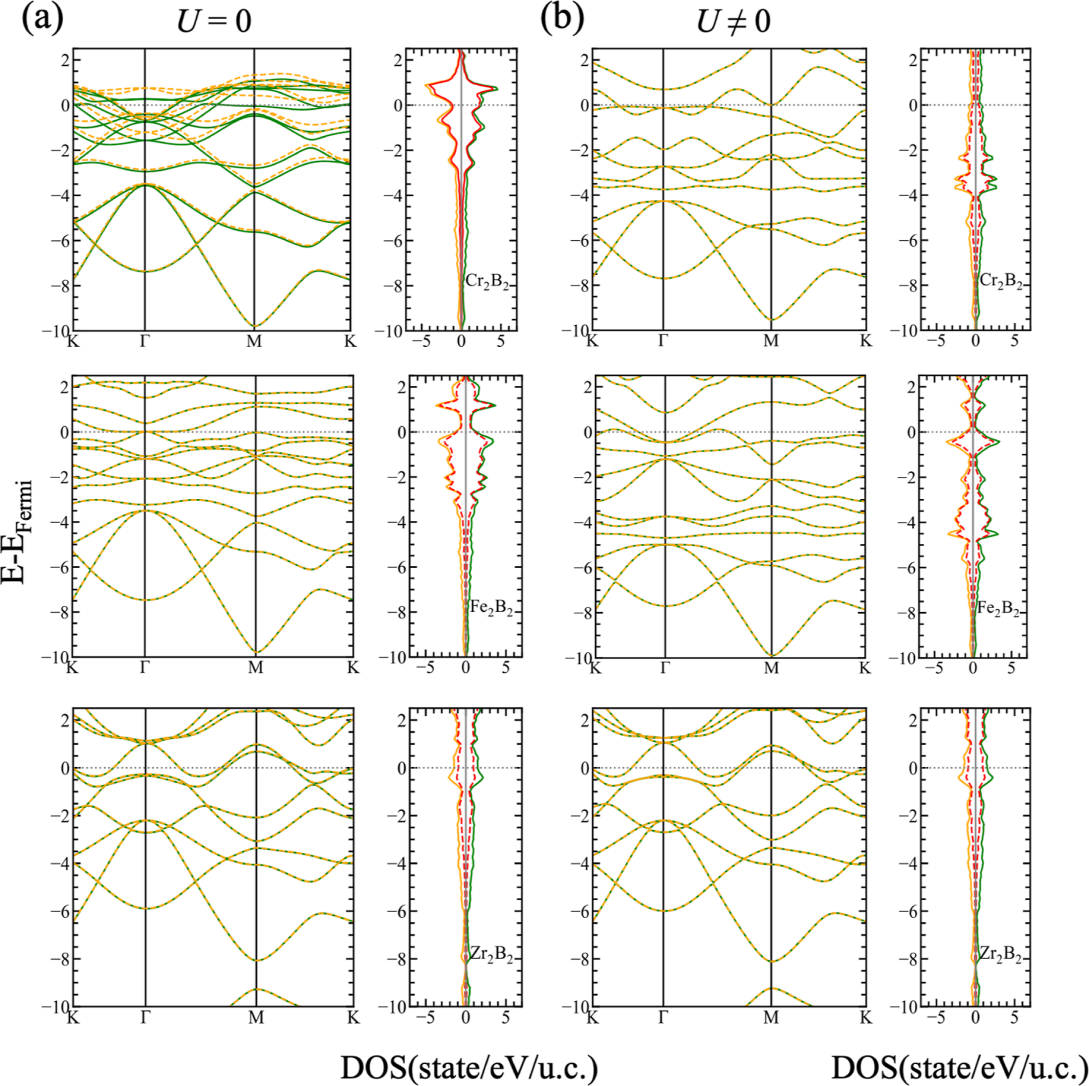
Comparison between the electronic band structure and DOS for the majority (green) and minority (orange) spins of the *P*6/*mmm* structures of Cr_2_B_2_, Fe_2_B_2_, and Zr_2_B_2_ using (a) DFT and (b) DFT+*U*. The dotted red line indicates the contribution of the TM-d orbitals.

The total DOS predicts the FM ground states for ortho-Cr_2_B_2_ and ortho-Fe_2_B_2_ with an asymmetry at the Fermi level between the two spin channels that transforms into AFM, in the case of ortho-Fe_2_B_2_, when *U* is used in the calculation of the electronic properties. It is also observed that only one band is crossing the Fermi level, which lies in a minimum of the DOS, and curiously, the systematic research of Dou et al. reported that ortho-Cr_2_B_2_ is a FM metal, while the ortho-Fe_2_B_2_ monolayer is a typically AFM semiconductor.^17^ Taking this result into consideration, some modifications like the addition of functional groups like OH, F, and O would allow to tailor the electronic properties for subsequent applications.^17,43^

Figure 3 plots simultaneously the p orbitals of boron and the d orbitals of the TM for the ortho-structures. When *U* = 0, it is clear that the Fermi level is dominated by the d states of the metals, and the p states of boron are negligible. Deep in energies (approximately in a range between −8 and −2 eV) are found the p states of boron, which partially hybridize with the d states of the TM and demonstrate the existing interaction between the TM and B atoms. The introduction of *U* dramatically changes the electronic properties of the materials and switches ortho-Fe_2_B_2_ into an AFM ground state. Focusing on the latter, here the p states of the boron shift toward the Fermi level, increasing their importance. Zr_2_B_2_ behaves as a nonmagnetic material, and the consideration of its own small *U* value does not introduce remarkable changes. Around the Fermi level, the d orbitals of Zr predominate, but the p orbitals of Zr also become relevant, showing a nonperfect hybridization with a similar behavior.

**Figure 5:**
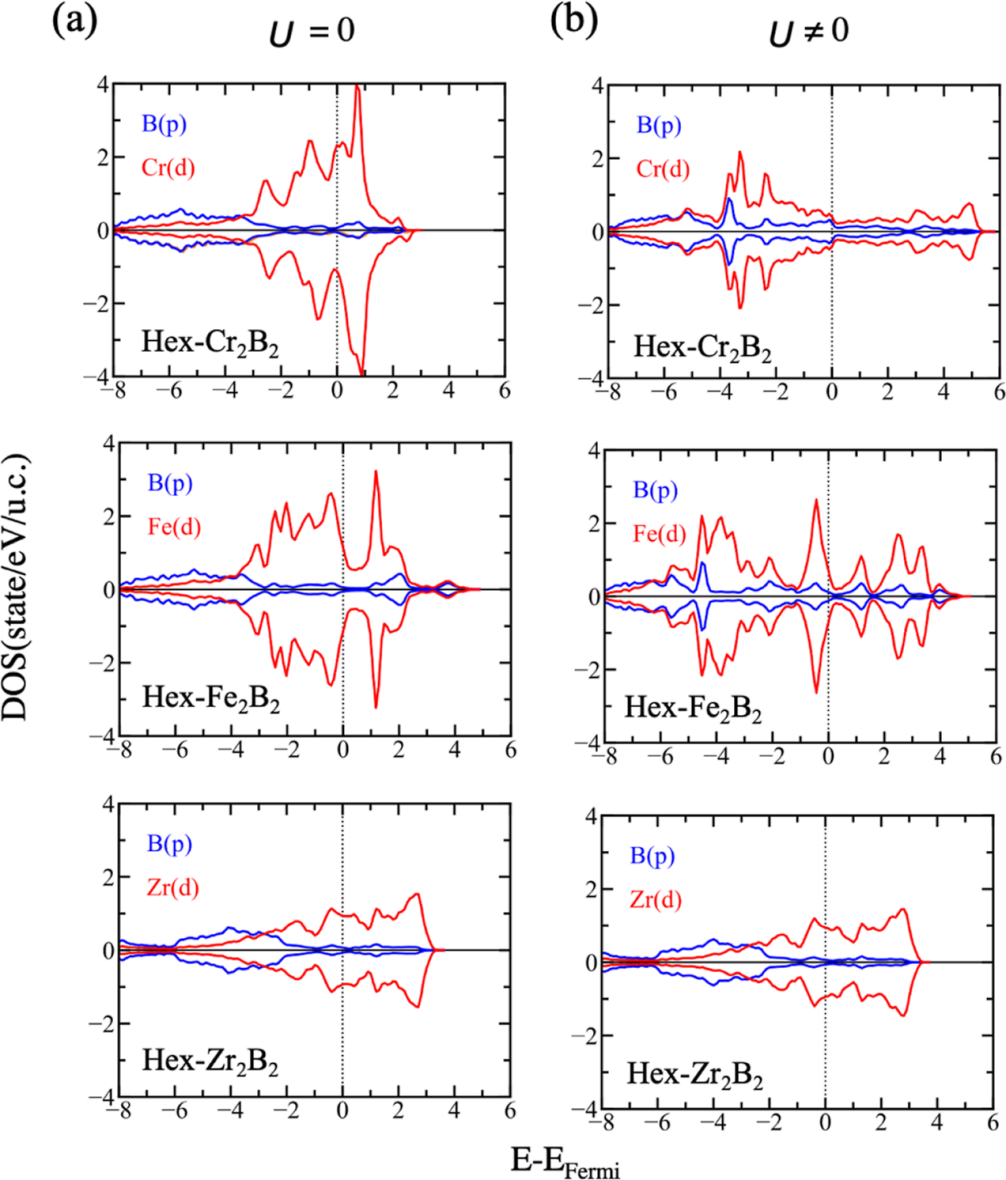
PDOS corresponding to the *d* states of the TM (red) and the p states of boron (blue) of the *P*6/*mmm* structures of Cr_2_B_2_, Fe_2_B_2_, and Zr_2_B_2_ using (a) DFT and (b) DFT+*U*.

The governing ground states of the hex-MBenes when *U* is considered are all AFM, which can be deduced from the band structures depicted in Figure 4, with the exception of hex-Zr_2_B_2_ which behaves again like nonmagnetic. Again, the p states of boron (Figure 5) are shifted toward the vicinity of the Fermi level, where, interestingly, the population of the d states is strongly diminished after including the correction.

**Figure 6:**
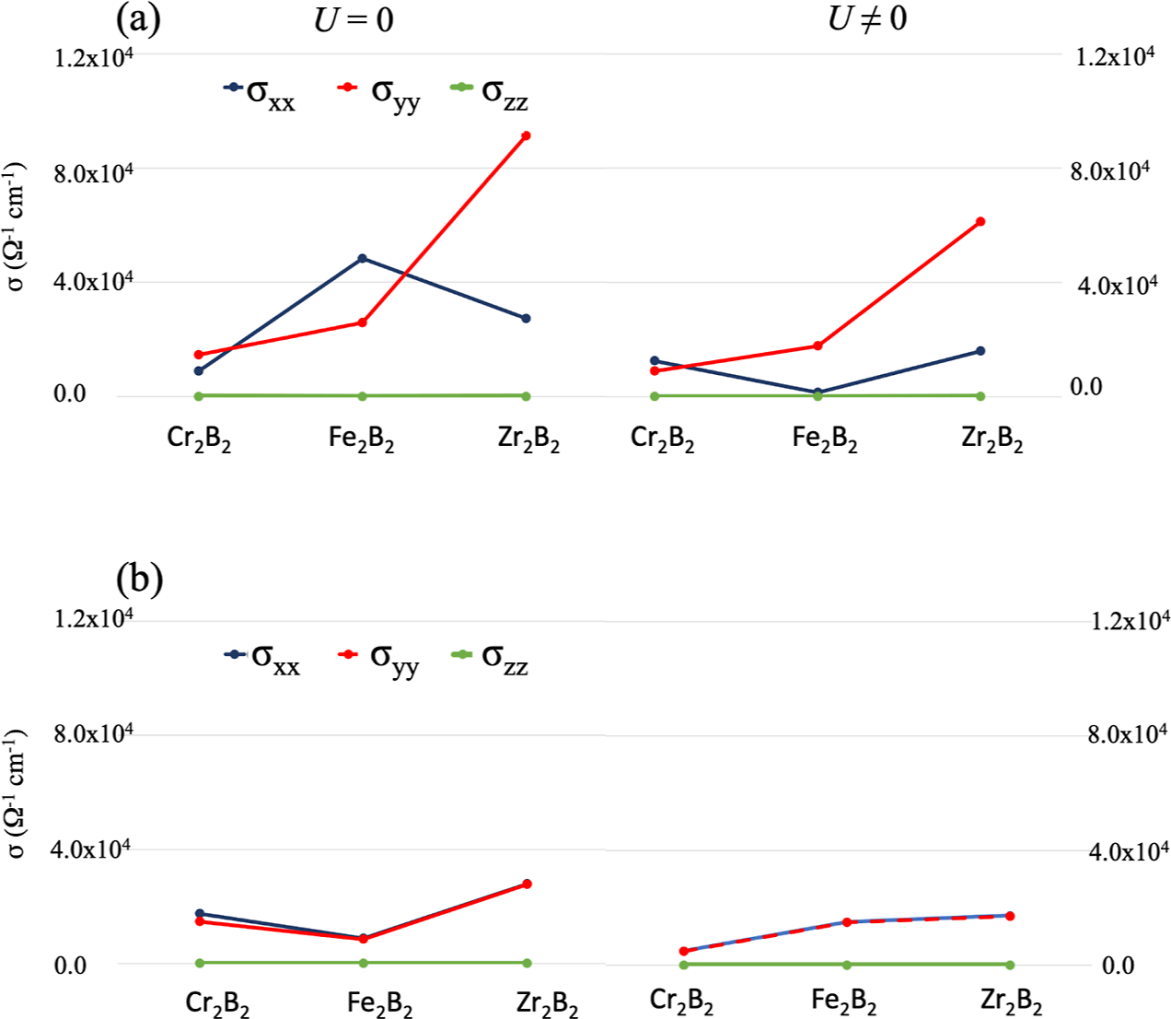
Components of the conductivity tensor for the (a) *Pmma* and (b) *P*6/*mmm* structures of Cr_2_B_2_, Fe_2_B_2_, and Zr_2_B_2_ considering *U* = 0 (left) and *U* ≠ 0 (right).

Generally speaking about ortho-MBenes, the components of the conductivity are strongly dependent on the in-plane directions, revealing their anisotropy (Figure 6). There are noticeable differences in the behavior of each MBene and also depending on the physical treatment adopted. While the conductivity takes place along the boron chain in ortho-Fe_2_B_2_, it is the opposite in Zr_2_B_2_. When *U* ≠ 0, on the other hand, it is for ortho-Cr_2_B_2_ that the conductivity happens along the boron chain instead. From the point of view of the conductivity, hex-MBenes are isotropic, presenting Zr_2_B_2_ the highest values among all.

### Magnetic Properties

The electron deficiency and low electronegativity of boron distinguish MBenes with intriguing magnetic properties. Some of the MBenes predicted as feasible exhibit robust metallic magnetism higher than 3 μ_B_ per TM atom and Curie temperatures over room temperature. Some studies suggest, moreover, that the critical temperatures can even be elevated under a careful selection of functional groups.^18,19,51^ To determine the MGS, two collinear configurations have been performed: one FM and another AFM, which consist of keeping the same spin alignment within each plane of metals. The results suggest a superexchange interaction because due to the large distances between the d orbitals, a direct overlap between them seems to be little realistic. Considering this, the d orbitals hybridize with the ligand atoms, that is, the 2p orbitals of boron, and hence, the magnetic interaction takes place between non-neighboring magnetic ions (TM) mediated by neighboring nonmagnetic ions (B). For such a magnetic interaction, the MGSs, which can be either FM or AFM, are empirically determined by the rules of Goodenough–Kanamori–Anderson^52–54^ based on the symmetry and the electron occupancy of the overlapping atomic orbitals. The magnetic moments of the boron atoms shown in Table 3 show that they are slightly polarized, demonstrating that magnetism in these cases is mediated by them.

**Table 3:** Charge Transfer from TM to B (Δ*q*), Total Magnetic Moment (μ_tot_) per Unit Cell, Magnetic Moment of the TM Atom (μ_TM/ion_), and Magnetic Moment of the Boron Atoms (μ_B/ion_) in Cr_2_B_2_, Fe_2_B_2_, and Zr_2_B_2_

	Δ*q*		μ_tot_ (μ_B_)		μ_TM/ion_ (μ_B_)		μ_B/ion_ (μ_B_)	
MBene	*U* = 0	*U* ≠ 0	*U* = 0	*U* ≠ 0	*U* = 0	*U* ≠ 0	*U* = 0	*U* ≠ 0
ortho-Cr_2_B_2_	–0.76	–0.82	2.56	6.39	1.03	2.79	–0.05	–0.22
hex-Cr_2_B_2_	–0.61	–0.63	0.63	0.00	0.31	±3.23	–0.01	0.00
ortho-Fe_2_B_2_	–0.37	–0.48	2.69	0.00	1.26	±2.31	–0.05	±0.08
hex-Fe_2_B_2_	–0.40	–0.41	0.00	0.00	±2.06	±2.59	0.00	0.00
ortho-Zr_2_B_2_	–1.17	–1.17	0.00	0.00	0.00	0.00	0.00	0.00
hex-Zr_2_B_2_	–0.86	–0.86	0.00	0.00	0.00	0.00	0.00	0.00

The two possible scenarios that emerge from the inclusion (or not) of the *U* parameter reach different conclusions, an effect found in a similar work^18^ where a thorough calculation with the increase in values of *U* produced a change in the MGSs of the same material. Excluding *U*, only hex-Fe_2_B_2_ adopted an AFM ground state, with an *E*_coh_ of the FM state being 46.84 meV smaller (Table 3) than that of the AFM state. However, ortho-Fe_2_B_2_ has a larger *E*_coh_ when the TM atoms arrange ferromagnetically, an effect observed before.^55^ Both orthorhombic and hexagonal structures of Cr_2_B_2_ result FM. Orthorhombic Cr_2_B_2_ and Fe_2_B_2_ with FM ordering possess magnetic moments over 2.5 μ_B_ per formula unit, indicating a suitable behavior as robust 2D magnets, but Zr_2_B_2_ exhibits nonmagnetic properties. The introduction of *U* causes the AFM states predominate over the FM states, and only ortho-Cr_2_B_2_ remains FM with a magnetic moment of 6.39 μ_B_, very close to the 6.10 and 6.49 μ_B_ values reported by Zhang et al.,^42^ after setting *U* = 4 eV for their calculations and *U* = 3 eV by Dou et al.,^17^ respectively, whereas the AFM behavior of ortho-Fe_2_B_2_ considering *U* ≠ 0 is in accordance to other works.^17,18^ The Bader charge analysis confirms that magnetism in these compounds arises from the d-electrons of the TM atoms. According to Table 3, the charge transference always happens from the TM to the boron atoms, with Zr being the metal for which the charge transference is larger. These values are in agreement to other works.^18,19^ Considering the Pauli exclusion principle together with Hund’s rules, the theoretical predicted magnetic moment of freestanding Cr, with electronic configuration [Ar] 3d^4^ 4s^2^, would be 4 μ_B_, whereas for iron, with electronic configuration [Ar] 3d^6^ 4s^2^, it will result in 3 μ_B_. As the calculated magnetic moments per metal ion are 2.79 and 3.23 μ_B_ for ortho- and hex-Cr_2_B_2_, respectively, and 2.31 and 2.59 μ_B_ for ortho- and hex-Fe_2_B_2_, we can conclude that the transference of one electron from the TM to the boron atom leads to the obtained magnetic moment and is, therefore, consistent with these results.

Attending to the results in Table 4, the large energy difference will increase the critical temperature beyond room temperature, a well-desired property for spintronic applications as mentioned above.

**Table 4:** MGS, Energy Difference, Δ*E*_FM-AFM_, between the FM and AFM Configurations, and Critical Temperature, *T*_c_, of Cr_2_B_2_, Fe_2_B_2_, and Zr_2_B_2_

	MGS		Δ*E*_FM-AFM_ (meV)		*T*_c_ (K)	
MBene	*U* = 0	*U* ≠ 0	*U* = 0	*U* ≠ 0	*U* = 0	*U* ≠ 0
ortho-Cr_2_B_2_	FM	FM	–104.38	–140.93	413	545
hex-Cr_2_B_2_	FM	AFM	–0.29	+46.61	1	180
ortho-Fe_2_B_2_	FM	AFM	–108.12	+540.66	418	2091
hex-Fe_2_B_2_	AFM	AFM	+46.84	+581.53	181	2249
ortho-Zr_2_B_2_	NM	NM				
hex-Zr_2_B_2_	NM	NM				

The exchange interaction, *J*_NN_, between the TM atoms at the nearest-neighbor (NN) positions can be evaluated with the energy difference Δ*E*_FM-AFM_ = *E*_FM_ – *E*_AFM_. It is well-known that the exchange energy for a system of interacting atomic moments can be described by the Heisenberg model
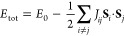
7where *E*_0_ is the total energy excluding spin–spin interactions and in our case *S*_*i*_ = *S*_*j*_ = *S*. For ferromagnetically or antiferromagnetically coupled TM ions at NN positions, −2*J*_NN_*S*^2^ = Δ*E*_FM-AFM_. Considering ferromagnetically or antiferromagnetically coupled TM ions at NN positions, −2*J*_NN_*S*^2^ = Δ*E*_FM-AFM_. On the other side, the critical temperature (Curie or Néel temperature), *T*_c_, is described in the mean field approximation (MFA) as
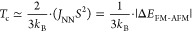
8

Although MFA usually overestimates the transition temperature for 2D magnets in ≈20% or, what is more, is dependent on the coordination number, however, this approximation is able to establish an upper limit of *T*_c_ at small computational cost.

Using eq 8, the resulting *T*_c_ values are collected in Table 4. As can be seen from the table, without the Hubbard correction, we obtain a considerably large value of *T*_c_ = 418 K for ortho-Fe_2_B_2_. However, as mentioned above, a more accurate investigation^19^ predicts an AFM ground state with *T*_c_ = 115 K. Interestingly, the calculations predict an AFM ground state for hex-Fe_2_B_2_ with *T*_c_ = 181 K. Adding the Hubbard correction, the MGS of ortho-Fe_2_B_2_ turns to AFM with an important energy difference. Other study^17^ computed even a higher difference for AFM ortho-Fe_2_B_2_, but using a smaller value of *U* = 3 eV, whereas their value for FM ortho-Cr_2_B_2_ is closer.

In this same direction, and having in mind that the current findings point out to high critical temperatures, much efforts are being done in finding MBenes for high Néel temperature AFM spintronics.^20^

## Conclusions

In the present work, the energetic, electronic, magnetic, and transport properties of selected MBenes have been systematically computed from two different perspectives (*U* = 0 and *U* ≠ 0) to be subsequently described, compared, and discussed. The findings reveal that the consideration or not of the Hubbard correction is not trivial and leads, in some cases, even to opposite results. Because the use of the parameter *U* is controversial, a thorough comparison with the experimental work is necessary to clarify which theoretical approach is the most suitable to describe the systems. The resulting values of *U* are typical of those found in previous studies for Cr_2_B_2_ and Fe_2_B_2_. In the particular case of Zr_2_B_2_, *U* is considerably smaller, and hence, this MBene maintains its properties unaffected with respect to the case of *U* = 0. The prevailing structures from an energetic point of view are orthorhombic for Cr_2_B_2_ and Fe_2_B_2_ and hexagonal for Zr_2_B_2_, whereas all of them become preferably hexagonal if high values for *U* are assumed. Also, the electronic properties are affected by such changes with a general decrease of states at the Fermi level, a situation reflected in the results for the conductivity. Up to this point, such discrepancies can influence the subsequent analysis for adsorption or catalytic processes, to give some examples. Moreover, the magnetic properties are strongly influenced by the value of *U*. The original MGS for *U* = 0, predominantly FM (except for hex-Fe_2_B_2_, with an AFM ground state), becomes mostly AFM after the inclusion of the corresponding Hubbard parameter (except for ortho-Cr_2_B_2_ which remains FM) reaching a high magnetic moment of 6.39 μ_B_ per formula unit, a desirable quality as a robust 2D magnet. Finally, the present calculations give rise to high critical temperatures, one of the reasons why MBenes are currently in the spotlight, opening the possibility of their use in room-temperature spintronics. In any case, it is clear that independently of the initial premises, all the MBenes are energetically stable, show a good conductive behavior, and, in some cases, are robust 2D magnets with high critical temperatures.
